# Enhancement of glucose and bone metabolism in ovariectomized rats fed with germinated pigmented rice with giant embryo (*Oryza sativa* L. cv. Keunnunjami)

**DOI:** 10.29219/fnr.v63.1612

**Published:** 2019-03-06

**Authors:** Soo Im Chung, Xingyue Jin, Mi Young Kang

**Affiliations:** Department of Food Science and Nutrition, Brain Korea 21 Plus, Kyungpook National University, Daegu, Korea

**Keywords:** pigmented giant embryo rice, Keunnunjami, germination, glucose, bone metabolism, menopause

## Abstract

**Background:**

Menopause induces various metabolic disorders due to the rapid decrease of the ovarian hormone estrogen. It is involved in increased risk of obesity, diabetes, dyslipidemia, and osteoporosis. The pigmented giant embryo cultivar is a promising food product for menopause-induced metabolic disorders.

**Objective:**

The effects of non-germinated and germinated Keunnunjami, a new blackish purple pigmented rice with a giant embryo, on glucose and bone metabolisms in ovariectomized rats were investigated.

**Design:**

The animals were fed with normal control diet (NC group) or control diet supplemented with either non-germinated Keunnunjami (KN group) or germinated Keunnunjami (GKN group) powder for 8 weeks.

**Results:**

The blood glucose and plasma insulin levels, adipokine concentrations, hepatic glucose-regulating enzyme activities, and bone resorption biomarker levels significantly decreased in KN and GKN groups compared to those of the control animals.

**Discussion:**

These findings illustrate that GKN group showed greater hypoglycemic activity and lower bone resorption than KN group, suggesting that germination could further improve the physiological property of Keunnunjami.

**Conclusion:**

Germinated Keunnunjami may have therapeutic potential against hyperglycemia and bone turnover imbalance caused by menopause.

## Popular scientific summary

- The Keunnunjami is pigmented giant embryo rice with a blackish purple pericarp which increased in amounts of bioactive compounds such as γ-Oryzanol, GABA, Tocols and Policosanol during germination.
- Germinated Keunnujami improved the glucose and bone metabolisms in ovariectomized rats via regulation of adipokine levels and glucose regulating enzyme activities.
- Germinated Keunnujami a potential functional foods in the prevention and management of menopause-induced metabolic disorders such as hyperglycemia and bone turnover imbalance.

## 

Keunnunjami is a new pigmented rice cultivar with blackish purple pericarp and a giant embryo that was developed in Korea through conventional breeding. It contains high amounts of natural antioxidant compounds and has strong antioxidant capacity (1, 2). A recent investigation on this rice cultivar revealed that it could modulate the lipid metabolism and lower the body fat in high-fat diet-fed mice (3). An instant rice made from a combination of this pigmented giant embryo cultivar and ordinary white rice has also been shown to reduce the lipid levels and body weight in high fat diet-fed mice (4). Pigmented rice is known to possess greater amounts of anthocyanins and phenolic compounds and higher nutritional value than non-pigmented rice (5, 6). Rice cultivar with an enlarged embryo has been reported to contain high levels of bioactive compounds, including γ-aminobutyric acid (GABA), γ-oryzanol, and tocopherol, which have hypolipidemic, hypoglycemic, and antioxidative properties (7–9).

A number of studies have shown that the amount of bioactive compounds in rice grain increases substantially during germination (10–12), a process of soaking the whole grains in water for a few days. Biochemical changes occur during this process, such as activation of dormant enzymes and release of free and bound materials, which results in the increase of bioactive compounds and nutrient bioavailability and absorption (13). Germinated cereal grains, including rice, wheat, and barley, contain significantly higher levels of GABA, γ-oryzanol, tocopherols, tocotrienols, and phenolics than non-germinated grains (12, 14, 15). Also, tocols and policosanol have the ability to suppress lipid peroxidation and may counteract cardiovascular disease (CVD) (16, 17). Moreover, germinated rice has been reported to have strong antidiabetic, hypolipidemic, and antioxidative activities (18). Dietary intake of germinated Keunnunjami (GKN) has been recently found to improve the lipid metabolism in ovariectomized rats (19).

In women, the permanent cessation of menstruation, known as menopause, induces various metabolic disorders due to the rapid decrease of the ovarian hormone estrogen (20). It is associated with increased risk of obesity, diabetes, dyslipidemia, and osteoporosis (21, 22). Ovariectomy, the surgical removal of ovaries, in animals mimics the estrogen-deficient condition in postmenopausal women. Hence, ovariectomized animals, particularly rats, have been widely used as postmenopausal-like animal model for investigating the metabolic dysfunctions caused by menopause. With the recent findings that GKN rice could lower the risk of dyslipidemia in ovariectomized rats (19), the present study was carried out to further explore the therapeutic potential of this pigmented giant embryo cultivar against menopause-induced metabolic disorders. The study aims to determine the effect of non-germinated and GKN rice on the glucose profile and bone turnover biomarkers in ovariectomized rats.

## Methods

### Preparation of germinated and non-germinated rice samples

Dehusked whole grains of Keunnunjami, which were grown in May–October 2014 in Dangjin, Chungcheongnam-do, South Korea, were obtained from the Department of Agricultural Science, Korea National Open University. They were germinated using the methods described by Wu et al. (22) with slight modifications. The grains (50 g) were washed twice with distilled water and placed evenly in a tray overlaid with cotton pads and cheesecloth and added with 100 mL distilled water. The tray was covered with a clean plastic wrap with holes to allow for ventilation and incubated at 30°C in an oven for 72 h. The grains were regularly checked every 12 h to ensure there was no foul odor and fungal growth. The germinated grains were then dried at 50°C for 2 h, ground and pulverized (200–300 μm) using a grinding machine (HMF-3250S, Hanil Electronics, Seoul, Korea), packed in hermetically sealed Ziploc plastic bags, and stored at −20°C until further analysis. For the non-germinated samples, the rice grains (50 g) were washed, dried, ground, and stored using the same method as described above. The germinated and non-germinated rice powders were analyzed for their proximate compositions using the methods of Association of Official Analytical Chemists (AOAC) (23) and for their bioactive compounds γ-oryzanol, GABA, tocols (tocopherols and tocotrienols), and policosanol following previously described methods (24–27). The results are shown in Table 1. All chemicals and standards used in this study were of analytical grade and purchased from Merck KGaA, Darmstadt, Germany, and Sigma-Aldrich, St. Louis MO, USA.

### Animals and diets

Female ovariectomized Sprague–Dawley rats (*n* = 30, 10 weeks old, 230 g each) were purchased from Central Laboratory Animal Inc. (Seoul, Korea). The current study protocol was approved by the Ethics Committee of Kyungpook National University for animal studies (approval no. 2015-0087). Each animal was housed in a hanging stainless steel cage in a room (25±2°C, 50% relative humidity) with 12/12 h light–dark cycle and fed initially with pelletized chow diet and distilled water ad libitum for 1 week. The animals were then randomly divided into three dietary groups (*n* = 10): normal control diet (NC) and NC diet supplemented with either 20% (w/w) non-germinated Keunnunjami (KN) or GKN rice powder. The composition of the experimental diet (Table 2) was based on the American Institute of Nutrition 93 maintenance (AIN-93M) (28). The rats were fed for 8 weeks and allowed free access to distilled water. At the end of the experimental period, they were anesthetized with carbon dioxide by inhalation following a 12-h fast. The blood samples were drawn from the inferior vena cava into a heparin-coated tube and centrifuged at 1,000 ***g*** for 15 min at 4°C to obtain the plasma. The liver, heart, kidney, and white adipose tissues (perirenal and inguinal) were removed, rinsed with physiological saline, weighed, and stored at −70°C until analysis.

**Table 1:** Proximate composition and bioactive components of Keunnunjami rice powder

Component	Non-germinated	Germinated
Proximate composition (% dry basis)		
Carbohydrates	76.20 ± 1.62*	56.22 ± 1.24
Protein	7.21 ± 0.38*	6.01 ± 0.47
Fat	2.62 ± 0.09	2.97 ± 0.75
Ash	1.36 ± 0.05	1.17 ± 0.06
Moisture	12.61 ± 0.91	33.63 ± 1.78*
Bioactive compound (mg/100 g rice)		
γ-Oryzanol	29.05 ± 1.58	44.89 ± 2.09*
GABA	99.69 ± 4.29	1201.57 ± 14.35*
Tocols	0.22 ± 0.01	0.41 ± 0.05*
Policosanol	22.25 ± 1.98	28.98 ± 1.47*

Values are means ± SE (n =3).

* indicates significant difference (*p* < 0.05) between germinated and non-germinated samples.

**Table 2:** Compositions of experimental diets (%)

Component	NC	KN	GKN
Casein	14.0	12.4	12.2
Sucrose	10.0	10.0	10.0
Dextrose	15.5	15.5	15.5
Corn starch	46.6	28.8	29.3
Cellulose	5.00	5.00	5.00
Soybean oil	4.00	3.40	3.10
Mineral mix	3.50	3.50	3.50
Vitamin mix	1.00	1.00	1.00
L-Cystine	0.18	0.18	0.18
Choline bitartrate	0.25	0.25	0.25
KN		20.0	
GKN			20.0

Total	100	100	100
Kcal	380	380	380

NC, normal control diet (AIN-93M); KN, normal diet + non-germinated Keunnunjami rice powder; GKN, normal diet + germinated Keunnunjami rice powder.

### Determination of glucose profile and plasma adipokine concentrations

The blood glucose and plasma insulin levels were measured using Accu-Chek Active Blood Glucose Test Strips (Roche Diagnostics, Indianapolis, IN, USA) and enzyme-linked immunosorbent assay (ELISA) kits (TMB Mouse Insulin ELISA kit, Shibayagi, Gunma, Japan), respectively. The level of hepatic glycogen was determined using the anthrone-H_2_SO_4_ method with glucose as standard (29). The homeostasis model assessment of insulin resistance (HOMA-IR) index was calculated using the equation described by Vogeser et al. (30). The following plasma adipokines were analyzed using commercial assay kits: adiponectin (Shibayagi), leptin (Cayman Chemical, Ann Arbor, MI, USA), resistin (B-Bridge International, Osaka, Japan), and tumor necrosis factor (TNF)-α (Abcam, Cambridge MA, USA).

### Analysis of hepatic glucose-regulating enzymes

The liver tissue was homogenized in a buffer solution containing triethanolamine, Ethylenediaminetetraacetic acid (EDTA), and dithiothreitol and centrifuged at 1,000 ***g*** at 4°C for 15 min (31). The pellet was discarded and the supernatant was centrifuged at 10,000 ***g*** at 4°C for 15 min. The resulting precipitate served as the mitochondrial fraction and the supernatant was further centrifuged at 105,000 ***g*** at 4°C for 1 h. The resulting precipitate and supernatant served as the microsome and cytosol fractions, respectively. The protein content was measured using Bradford protein assay (32). The phosphoenolpyruvate carboxykinase activity was analyzed using the method of Bentle and Lardy (33). The absorbance of the assay mixture was measured at 340 nm. The glucose-6-phosphatase activity was analyzed using the method of Alegre et al. (34). The reaction mixture was incubated at 37°C for 4 min, and the change in absorbance at 340 nm was recorded. The glucokinase activity was determined using the method of Davidson and Arion (35). The reaction mixture was incubated at 37°C for 10 min, and the change in absorbance at 340 nm was recorded. The enzyme activities were expressed as μmol/min/mg protein.

### Analysis of bone metabolism biochemical markers

The levels of 17-β-estradiol, intact parathyroid hormone (PTH), osteocalcin, N-terminal telopeptide of type 1 collagen (NTx-1), and C-terminal telopeptide of type 1 collagen (CTx-1) were determined using commercial assay kits (MyBiosource, San Diego, CA, USA). The calcium and alkaline phosphatase (ALP) contents were measured using Ca and ALP assay kits (Cobas, Tokyo, Japan), respectively.

### Statistical analysis

The data were evaluated by one-way Analysis of Variance (ANOVA) using the Statistical Package for Social Sciences (SPSS) software program version 19.0, and the differences between the means were assessed using Tukey’s test. Independent *t*-test was used to assess the difference between the germinated and non-germinated rice samples. Statistical significance was considered at *p* < 0.05.

## Results

### Body weight gain

At the end of the experimental period, the body weight gain was lowest in GKN group (132 g), followed by KN rats (154 g) (Table 3). The NC group showed the highest weight gain (174 g). The GKN group exhibited significantly lower feed intake and feed efficiency ratio than the control group. The white adipose tissue weight was highest in NC group (11.5 g) and lowest in GKN group (8.8 g). Both the KN and GKN groups showed significantly lower weights of the liver, heart, and kidney than the control group.

**Table 3:** Body weight gain, feed intake, and weights of adipose tissue and internal organs in ovariectomized rats fed with germinated Keunnunjami rice powder

Parameter	NC	KN	GKN
Initial weight (g)	232.14 ± 2.98	230.32 ± 1.21	231.98 ± 1.52
Final weight (g)	405.88 ± 5.62^c^	381.87 ± 4.51^b^	359.14 ± 5.84^a^
Weight gain (g)	174.29 ± 5.63^c^	153.57 ± 3.41^b^	131.55 ± 4.25^a^
Feed intake (g/day)	24.74 ± 1.32^b^	23.31 ± 1.45^ab^	21.87 ±1.16^a^
Feed efficiency ratio	0.11 ± 0.00^c^	0.10 ± 0.00^b^	0.08 ± 0.00^a^
White adipose tissue weight (g)	11.54 ± 0.19^c^	9.44 ± 0.11^b^	8.81 ± 0.05^a^
Organ weight (g)			
Liver	2.71 ± 0.01^c^	2.62 ± 0.01^b^	2.47± 0.01^a^
Heart	0.24 ± 0.00^b^	0.24 ± 0.00^b^	0.23 ± 0.00^a^
Kidney	0.40 ± 0.01^b^	0.38 ± 0.00^a^	0.37 ± 0.01^a^

Values are means ± SE (*n* =10).

a-c Means in the same row not sharing a common superscript are significantly different at *p* < 0.05. NC, normal control diet (AIN-93M); KN, normal diet + non-germinated Keunnunjami; GKN, normal diet + germinated Keunnunjami.

### Glucose profile

The NC group exhibited the highest final blood glucose level (7.01 mmol/L) and plasma insulin level (7.98 mU/L) and the lowest hepatic glycogen content (96.6 mg/g) among the animal groups (Table 4). The GKN group, on the other hand, showed the lowest levels of glucose (5.24 mmol/L) and insulin (3.59 mU/L) and the highest amount of glycogen (163 mg/g). The HOMA-IR index, an indicator of insulin resistance, was lowest in GKN group (0.81) and highest in the control group (1.61).

**Table 4:** Glucose profile, adipokine level, and glucose-regulating enzyme activity in ovariectomized rats fed with germinated Keunnunjami rice powder

Parameter	NC	KN	GKN
Initial blood glucose (mmol/L)	5.03 ± 0.03	5.10 ± 0.04	5.14 ± 0.02
Final blood glucose (mmol/L)	7.01 ± 0.05^c^	5.59 ± 0.03^b^	5.24 ± 0.03^a^
Plasma insulin (mU/L)	4.98 ± 0.12^c^	4.01 ± 0.04^b^	3.59 ± 0.02^a^
Hepatic glycogen (mg/g liver)	96.58 ± 5.46^a^	111.69 ± 3.78^b^	163.58 ± 5.17^c^
HOMA-IR index	1.61 ± 0.00^c^	1.00 ± 0.02^b^	0.81 ± 0.00^a^
Plasma adipokine			
Adiponectin (ng/mL)	0.24 ± 0.03^a^	0.45 ± 0.03^b^	0.69 ± 0.06^c^
Leptin (ng/mL)	4.82 ± 0.24^b^	4.23 ± 0.32^a^	4.32 ± 0.25^a^
Resistin (ng/mL)	33.25 ± 0.47^c^	22.88 ± 1.43^b^	18.25 ± 1.05^a^
Tumor necrosis factor-α (μg/mL)	9.84 ± 0.68^c^	7.65 ± 0.68^b^	3.81 ± 0.22^a^
Hepatic glucose-regulating enzymes (μmol/min/mg protein)			
Phosphoenolpyruvate carboxykinase	3.67 ± 0.55^c^	1.21 ± 0.08^b^	0.89 ± 0.06^a^
Glucose-6-phosphatase	74.55 ± 2.62^c^	55.62 ± 1.27^b^	39.25 ± 1.21^a^
Glucokinase	1.56 ± 0.11^a^	4.87 ± 0.22^b^	5.32 ± 0.58^b^

Values are means ± SE (*n* =10).

a-c Means in the same row not sharing a common superscript are significantly different at *p* < 0.05. NC, normal control diet (AIN-93M); KN, normal diet + non-germinated Keunnunjami; GKN, normal diet + germinated Keunnunjami; HOMA-IR, homeostasis model of insulin resistance = (fasting insulin x fasting glucose)/22.5.

### Plasma adipokines

The NC group showed significantly lower levels of adiponectin (0.24 ng/mL), leptin (4.82 ng/mL), resistin (33.2 ng/mL), and TNF-α (9.84 μg/mL) compared to other animal groups (Table 4). Between the two Keunnunjami-fed groups, the GKN mice exhibited significantly higher adiponectin level (0.69 ng/mL) and lower resistin (18.2 ng/mL) and TNF-α (3.81 μg/mL) contents.

### Hepatic glucose-regulating enzyme activities

The activities of hepatic phosphoenolpyruvate carboxykinase and glucose-6-phosphatase were highest in NC rats (3.67 and 74.6 μmol/min/mg protein, respectively) and lowest in GKN group (0.89 and 39.2 μmol/min/mg protein, respectively) (Table 4). On the other hand, the KN and GKN groups showed significantly lower glucokinase activity (4.87 and 5.32 μmol/min/mg protein, respectively) than the NC group (1.56 μmol/min/mg protein).

### Biomarkers of bone metabolism

The 17-β-estradiol level was highest in GKN group (789 pg/mL), followed by KN group (614 pg/mL) and then NC group (55 pg/mL) (Table 5). The amounts of intact PTH, NTx-1, and CTx-1 were highest in NC group (24.0 pg/mL, 85.6 ng/mL, and 7.55 ng/mL, respectively) and lowest in GKN group (15.9 pg/mL, 47.2 ng/mL, and 0.85 ng/mL, respectively). The calcium and osteocalcin levels were not significantly different among the groups. The ALP content, on the other hand, was below 0.50 μg/L in all animal groups.

**Table 5:** Biomarkers of bone metabolism in ovariectomized rats fed with germinated Keunnunjami rice powder

	NC	KN	GKN
17-β-estradiol (pg/mL)	558.25 ± 28.21^a^	614.42 ± 11.32^b^	788.87 ± 9.95^c^
Intact PTH (pg/mL)	24.01 ± 1.02^c^	20.52 ± 1.22^b^	15.87 ± 1.07^a^
Calcium (mg/dL)	10.25 ± 1.21	10.38± 0.93	10.19 ± 1.21
Osteocalcin (ng/mL)	13.47 ± 1.03	12.68 ± 1.14	12.45 ± 1.7
Alkaline phosphatase (μg/L)	<0.50 ± 0.00	<0.50 ± 0.00	<0.50 ± 0.00
NTx-1 (ng/mL)	85.61 ± 3.87^c^	55.26 ± 1.87^b^	47.2 ± 1.35^a^
CTx-1 (ng/mL)	7.55 ± 0.13^c^	2.34 ± 0.12^b^	0.85± 0.05^a^

Values are means ± SE (*n* =10).

a-c Means in the same row not sharing a common superscript are significantly different at *p* < 0.05. NC, normal control diet (AIN-93M); KN, normal diet + non-germinated Keunnunjami; GKN, normal diet + germinated Keunnunjami; PTH, parathyroid hormone; NTx-1, N-terminal telopeptide of type 1 collagen; CTx-1, C-terminal telopeptide of type 1 collagen.

## Discussion

Menopause is known to induce several metabolic disorders, including diabetes and osteoporosis (20, 21). In the present study, the effect of GKN, a new blackish purple pigmented rice with a giant embryo, on the glucose profile and bone turnover in postmenopausal-like model of ovariectomized rats was investigated. Results showed that dietary feeding of GKN and KN powders significantly reduced the body weight, body fat, blood glucose and plasma insulin levels, and HOMA-IR index and increased the hepatic glycogen content in ovariectomized rats. This improvement in the glucose profile in rice-fed animal groups was possibly influenced by the regulation of adipokine production and glucose-regulating enzyme activities. Both the KN and GKN groups exhibited markedly higher adiponectin content and glucokinase enzyme activity than the NC group. On the other hand, they showed considerably lower levels of leptin, resistin, and TNF-α and activities of phosphoenolpyruvate carboxykinase and glucose-6-phosphatase enzymes. The adipokine adiponectin could induce insulin-sensitizing effects and its elevated level has been associated with improved insulin sensitivity and glucose tolerance and could protect women against the development of diabetes after menopause (36, 37). The adipokines leptin, resistin, and TNF-α are also involved in lipid and glucose metabolisms, and their enhanced expressions have been linked with the development of diabetes and obesity (37, 38). Likewise, the enhanced activities of phosphoenolpyruvate carboxykinase and glucose-6-phosphatase enzymes, which regulate gluconeogenesis and hepatic glucose output, could lead to increased production of glucose (39, 40). On the other hand, the enhanced activity of glucokinase enzyme, which is involved in glucose homeostasis, has been associated with increased amount of glycogen and reduced level of blood glucose (41).

Results of the present study also showed that diet supplementation of Keunnunjami powder markedly increased the amount of 17-β-estradiol and substantially reduced the levels of intact PTH, NTx-1, and CTx-1 in ovariectomized rats, suggesting an improved bone metabolism in these animals. The 17-β-estradiol is the most potent form of estrogen hormone, which plays a vital role in the regulation of bone metabolism (42). The rapid decrease of estrogen after menopause is believed to be responsible for the development of various metabolic syndromes in women (20). Previous studies have shown that administration of 17-β-estradiol decreased the bone turnover rate and prevented bone loss in postmenopausal women (43–45). The intact PTH, NTx-1, and CTx-1 are biochemical markers of bone resorption, and their reduced levels indicate decreased bone turnover. The rapid rate of bone loss and increased risk of bone fracture in postmenopausal women resulted from increased bone resorption and imbalanced bone turnover (21, 42).

Previous studies have revealed that rice cultivars with colored pericarp could lower the body weight, enhance the glucose metabolism, and increase the antioxidant status in both animals and humans (46–48). Rice cultivars with giant embryo have also been shown to reduce the body weight and glucose level in diabetic rats (49). Keunnunjami rice, a pigmented cultivar with a giant embryo, is rich in bioactive compounds, including γ-oryzanol, GABA, tocopherol, tocotrienol, and policosanol, which have antihyperlipidemic, anti-obesity, antidiabetic, and antioxidative properties (7, 50, 51). Chung et al. (1) recently reported that Keunnunjami rice contains relatively high amounts of phenolics and flavonoids and has strong antioxidant capacity. Moreover, it has been reported that dietary feeding of this pigmented giant embryo rice reduced the body weight and improved the lipid metabolism in high fat diet-fed mice (3).

Between the two Keunnunjami rice-fed groups, the GKN rats showed significantly greater hypoglycemic effect and bone metabolism-improving action. Lo et al. (19) reported that diet supplementation of GKN could also improve the lipid profile and lower the risk of dyslipidemia in ovariectomized rats. Germination increases the nutritional value and bioactive compounds in cereal grains (12, 14) due to the various biochemical changes that occur during this process, such as breaking down of cell wall, which results in the release of free and bound materials, and activation of dormant enzymes involved in the synthesis of bioactive components (10, 13). In the present study, the GKN rice contained substantially higher levels of γ-oryzanol, GABA, tocols, and policosanol than the non-germinated rice. In particular, the GABA content increased by 12-fold after germination. GABA has been found to be the most generated component during germination, and it has anti-obesity, antidiabetic, and anticancer properties (10, 52). The pathogenesis of osteoporosis is associated with oxidative stress (53, 54), and it has been suggested that menopause-induced bone loss could be prevented by treatment with antioxidants (55). Recent studies have also shown that an antioxidant-based dietary approach could be beneficial in the prevention and treatment of osteoporosis in postmenopausal women (56, 57). It is therefore possible that the strong hypoglycemic effect and enhanced bone metabolism observed in GKN rats is due to the increased amounts of several bioactive compounds, which have antidiabetic and antioxidative properties, in GKN rice. In conclusion, the giant embryo pigmented rice cultivar, Keunnunjami, markedly reduced the body weight and improved the glucose and bone metabolisms in ovariectomized rats via regulation of adipokine levels and glucose-regulating enzyme activities. These hypoglycemic and bone metabolism-improving effects of Keunnunjami were further enhanced after germination for 72 h, which may have been possibly due to the increased amounts of bioactive compounds GABA, γ-oryzanol, tocol, and policosanol during germination. The results suggest that GKN may be useful in the prevention and management of menopause-induced metabolic disorders such as hyperglycemia and bone turnover imbalance.
